# Temporal trends in children pertussis burden in China and worldwide from 1990 to 2023: An analysis of the Global Burden of Disease Study 2023

**DOI:** 10.1371/journal.pone.0354164

**Published:** 2026-07-27

**Authors:** Beinan Xu, Jingjing Gu, Xiaolin Ye, Dongling Liu, Shangfu Xie, Yuanlin Lv, Yixuan Mou, Wei Wu

**Affiliations:** 1 The First School of Clinical Medicine, Zhejiang Chinese Medical University, Hangzhou, China; 2 School of Basic Medical Sciences, Zhejiang Chinese Medical University, Hangzhou, China; Gabriele d’Annunzio University of Chieti and Pescara: Universita degli Studi Gabriele d’Annunzio Chieti Pescara, ITALY

## Abstract

**Objective:**

To evaluate the burden trends of children pertussis worldwide and in China from 1990 to 2023, and predict the disease burden for the next 10 years using a secondary analysis of Global Burden of Disease (GBD) 2023 data.

**Methods:**

The primary metrics were mortality, disability-adjusted life - years (DALYs), prevalence, and incidence. A join – point regression model was used to estimate the average annual percentage change (AAPC). The autoregressive integrated moving average (ARIMA) model was used to predict the disease burden of pertussis from 2024 to 2033, and its adequacy was confirmed by the Ljung‑Box test.

**Results:**

From 1990 to 2023, childhood pertussis mortality, DALYs, prevalence, and incidence declined sharply worldwide and in China. The global mortality rate in infants aged 1–5 months decreased by 57.7%, from 108.09 per 100,000 in 1990 to 45.73 per 100,000 population in 2023. Global age-standardized rates for both sexes, as well as those in all SDI regions, also showed steady downward trends. The rates of all four metrics peaked among infants aged 1–5 months in China and declined steadily with increasing age. For this infant group, the APC of pertussis prevalence and incidence in China decreased significantly during three different periods (1995–2004, 2009–2018, 2018–2021), but rose in two intervals (2004–2009, 2021–2023). ARIMA model projections revealed that age-standardized mortality and DALY rates will rise over the next decade, while prevalence and incidence will continue to decline. Notably, prevalence and incidence among infants aged 1–5 months are expected to increase slightly, rising from 277.59 and 2,026.43 per 100,000 in 2024 to 284.04 and 2,073.46 per 100,000 in 2033, respectively.

**Conclusion:**

These findings underscore the disproportionate and persistent burden of pertussis in infants, indicating that prevention and treatment strategies should be adjusted to prevent further increase.

## Introduction

Although global vaccine coverage is high, pertussis has resurfaced as the primary vaccine-preventable source of childhood morbidity worldwide [1,2]. Between 1990 and 2021, global childhood pertussis incidence, mortality, and DALYs had fallen steadily. Nevertheless, infants < 1 year continue to bear disproportionately high burden, making robust vaccination, prompt diagnosis, and complication prevention more critical than ever [3]. Data from France (2008–2020) confirm that pertussis remains a persistent public health challenge, especially during the first year of life, and suggest that widespread adoption of maternal vaccination could reduce this risk [2]. In low socio-demographic index (SDI) settings such as Somalia, pertussis still imposes a heavy burden, underscoring the urgent need for sustained financial and medical support [3].

China, once considered a low-incidence country, is undergoing a similar epidemiologic shift. Crucially, this national resurgence is no longer inferred from limited regional observations but is solidly grounded in nationwide notifiable disease surveillance data and systematic multinational evidence [4]. In southwestern China, pertussis has replaced traditional acute respiratory infections, such as measles, rubella, and mumps, as the predominant respiratory illness [5]. Nationwide, both pertussis incidence and associated deaths have increased dramatically [6,7]. The sharp rise in pertussis cases reflects multiple interacting factors, including the progressive evolution of *Bordetella pertussis* towards enhanced pathogenicity, increased surveillance sensitivity, improved diagnostic capacity, and the broader impact of the COVID-19 pandemic [5–7].

Despite these concerning trends, several critical knowledge gaps remain. First, current pertussis case definitions likely underestimate the true disease burden, and many infections escape detection in routine surveillance systems [8]. Second, country‑specific burden estimates, particularly for China, are urgently needed to guide the optimal timing of booster doses, the implementation of maternal immunization programs, and the precise allocation of limited public health resources. To date, however, no comprehensive study has systematically quantified the recent pertussis burden among Chinese children while simultaneously comparing it with global trends and assessing the sensitivity of current surveillance.

To address these gaps, we quantify the contemporary pertussis burden globally and among Chinese children, delineate age-specific incidence and mortality, and assess the sensitivity of current surveillance. This study aims to provide the evidence-based foundation for formulating targeted vaccination strategies and curbing the resurgence of pertussis in China.

## Methods

### Data source

Data for this study were extracted from the Global Burden of Diseases, Injuries, and Risk Factors Study (GBD) 2023, a systematic analysis that quantifies the burden of 375 diseases and injuries, the risk-attributable burden of 88 risk factors, and 292 specific causes of death across 204 countries and territories and 660 subnational locations from 1990 to 2023. The dataset integrates over 55,000 data sources, including vital registries, surveillance systems, household surveys, cohort studies, and randomized controlled trials-employs standardized modeling tools and comparative risk assessment to generate comprehensive, age- and sex-specific estimates of incidence, prevalence, mortality, and healthy life expectancy [9,10]. The Socio-demographic Index (SDI) is a composite indicator of development status strongly correlated with health outcomes. It is the geometric mean of 0–1 indices of total fertility rate under the age of 25, mean education for those ages 15 and older, and lag distributed income per capita. A location with an SDI of 0 would have a theoretical minimum level of development relevant to health, while a location with an SDI of 1 would have a theoretical maximum level [11]. The 2023 GBD analyses customarily divide countries into Low SDI, Low-middle SDI, Middle SDI, High-middle SDI.

The data related to children pertussis in this study were sourced from the Global Health Data Exchange (GHDx) and its affiliated tools (http://ghdx.healthdata.org/gbd-resultstool). In the GBD 2023 framework, pertussis cases were identified using ICD-10 code A37. Age‑specific estimates were systematically extracted for the following pediatric groups: 1–5 months, 6–11 months, 12–23 months, 2–4 years, 5–9 years, 10–14 years, and 15–19 years. We utilized the GBD tool to extract data on the deaths, disability-adjusted life-years (DALYs), prevalence, and incidence rates in China, SDI, and the worldwide from 1990 to 2023. The institutional ethics committee granted an exemption for this study, as it did not require approval, given that the data from the 2023 GBD are publicly available. This study adhered to the guidelines for accurate and transparent health assessment reporting.

### Statistical analysis

We screened the deaths (mortality), DALYs, prevalence, incidence and corresponding age-standardized rates of mortality (ASMR), age-standardized DALY rates (ASDR), age-standardized prevalence rates (ASPR), and age-standardized incidence rates (ASIR) of children pertussis in China and worldwide from the GBD database.

The average annual percentage change (AAPC) and corresponding 95% uncertainty interval (95% UI) were calculated using Joinpoint software (National Cancer Institute, Rockville, MD, USA) to determine the burden trend of the disease [12]. The AAPC was calculated as 100 × (exp(β) − 1), with the 95% UI derived directly from the model. An increasing trend is indicated if the 95% UI of the AAPC estimate is entirely above zero; a decreasing trend is suggested if the UI is entirely below zero; and a stable trend is inferred if the UI includes zero.

We generated forecasts for 2024–2033 by combining a linear mixed-effects model, spline interpolation, and an autoregressive integrated moving average (ARIMA) model on residuals to estimate the burden of childhood pertussis [13]. Model adequacy was examined using the Ljung-Box Q test, with residuals considered white noise when p > 0.05. Predictive accuracy was quantified using the root mean square error (RMSE) and the mean absolute percentage error (MAPE). The optimal model was selected based on the Akaike information criterion (AIC) and the Bayesian information criterion (BIC). A sensitivity analysis was performed by initializing the ARIMA model with three alternative fixed orders: (0,1,0), (0,1,1), and (0,1,2). Regardless of the pre‑specified starting parameters, the auto.arima procedure consistently identified ARIMA(0,1,2) as the optimal specification, yielding identical regression coefficients, information criteria, and residual diagnostics. The consistency of the model outputs across different initial settings demonstrates the robustness of our forecasts and confirms that the results are not sensitive to minor changes in the pre‑set model orders.

All statistical analyses and graphics were performed using the R statistical software package. Statistical analyses were conducted using R software version 4.4.2 (http://www.R-project.org). A *p* value less than 0.05 was considered statistically significant.

### Ethics statements

This study utilized de-identified, publicly available data from the Global Burden of Diseases, Injuries, and Risk Factors Study (GBD) 2023 database. Therefore, this study was exempt from additional ethical approval and the requirement for informed consent.

## Results

### Childhood pertussis burden across the globe and SDI regions

Between 1990 and 2023, global childhood pertussis burden declined sharply across all indicators including deaths, DALYs, prevalence and incidence (Table 1). A key pattern emerges when distinguishing between the number of cases and the rate of disease. The number of global mortality cases was highest among children aged 2–4 years, dropping 60.6% from 65,820.86 (95% UI: 32,444.29−110,116.20) in 1990–25,953.68 (95% UI: 14,786.20−43,587.41) in 2023 (S1 Table). However, global mortality rate (a more direct measure of individual risk) was highest in infants aged 1–5 months, dropping 57.7% from 108.09 (95% UI: 57.63–183.55) per 100,000 population in 1990 to 45.73 (26.01–78.96) per 100,000 population in 2023. Adolescents aged 15–19 years had the lowest rates, falling from 0.26 (95% UI: 0.13–0.46) per 100,000 population in 1990 to 0.10 (95% UI: 0.06–0.17) per 100,000 population in 2023. This dissociation between highest count (older children) and highest rate (young infants) indicates that while total deaths are more numerous in the 2–4 years group, the per-capita risk faced by infants under 5 months is substantially greater. Adolescents aged 15–19 years had the lowest rates across all metrics (Table 2 and S2 Table).

**Table 1:** The rate of mortality and DALYs in pertussis burden for both genders between 1990 and 2023.

		1990		2023	
Location	Age	Deaths rate per 100,000 people (95% UI)	DALYs rate per 100,000 people (95% UI)	Deaths rate per 100,000 people (95% UI)	DALYs rate per 100,000 people (95% UI)
Global	Age-standardized	4.61 (2.40,8.08)	403.61 (210.99,704.74)	1.79 (1.03,2.94)	157.90 (91.16,257.53)
	1-5 months	108.09 (57.63,183.55)	9780.98 (5228.72,16569.58)	45.73 (26.01,78.96)	4151.59 (2380.45,7135.54)
	6-11 months	75.4 (39.01,129.57)	6792.94 (3539.03,11646.2)	33.49 (19.43,57.33)	3026.07 (1764.92,5152.46)
	12-23 months	50.81 (25.41,87.44)	4543.07 (2285.05,7794.5)	19.63 (11.4,32.26)	1763.85 (1030.35,2888.39)
	2-4 years	18.04 (8.89,30.18)	1578.34 (783.64,2634.57)	6.61 (3.76,11.1)	580.55 (333.2,970.24)
	5-9 years	6.18 (3.11,10.92)	517.82 (261.19,911.29)	1.92 (1.11,3.04)	161.5 (94.18,254.54)
	10-14 years	1.21 (0.63,2.14)	95.34 (49.96,167.46)	0.47 (0.27,0.78)	37.35 (21.61,61.72)
	15-19 years	0.26 (0.13,0.46)	18.87 (9.77,33.85)	0.10(0.06,0.17)	7.44 (4.26,12.44)
China	Age-standardized	1.76 (0.16,4.77)	153.85 (15.59,414.31)	0.13 (0.02,0.36)	12.25 (2.14,31.33)
	1-5 months	38.87 (3.30,111.84)	3531.05 (330.16,10080.19)	2.67 (0.29,7.49)	255.12 (42.14,688.90)
	6-11 months	32.09 (2.96,86.89)	2897.43 (289.93,7793.73)	1.71 (0.23,4.15)	164.76 (31.70,381.31)
	12-23 months	18.43 (1.78,47.71)	1654.86 (176.80,4248.43)	1.72 (0.21,4.60)	160.62 (27.21,414.26)
	2-4 years	6.37 (0.60,16.72)	559.82 (57.80,1458.93)	0.52 (0.07,1.33)	47.88 (8.29,119.61)
	5-9 years	2.54 (0.24,6.90)	212.55 (21.55,574.12)	0.19 (0.02,0.50)	16.87 (2.67,42.03)
	10-14 years	0.54 (0.05,1.58)	42.46 (4.1,123.19)	0.05 (0.01,0.13)	4.01 (0.62,10.44)
	15-19 years	0.12 (0.01,0.34)	8.45 (0.8,24.58)	0.01 (0,0.03)	0.81 (0.12,2.19)
High-middle SDI	Age-standardized	2.45 (1.03,5)	214.75 (91.52,433.97)	0.57 (0.25,1.16)	51.29 (23.51,102.66)
Middle SDI	Age-standardized	3.89 (1.78,7.56)	340.46 (157.21,657.02)	0.98 (0.47,1.79)	86.87 (42.31,155.91)
Low-middle SDI	Age-standardized	6.46 (2.28,13.57)	563.41 (201.89,1174.36)	1.54 (0.71,2.88)	135.37 (63.3,251.92)
Low SDI	Age-standardized	10.42 (5.04,17.12)	905.51 (440.69,1480.79)	3.59 (1.95,6.13)	314.42 (171.58,534.51)
India	Age-standardized	7.15 (0.9,16.89)	623.23 (82.83,1463.04)	1.07 (0.12,2.88)	94.2 (12.23,251.67)
Japan	Age-standardized	0.01 (0,0.01)	2.34 (1.56,3.53)	0 (0,0)	0.47 (0.32,0.69)

**Table 2:** The rate of prevalence and incidence in pertussis burden for both genders between 1990 and 2023.

		1990		2023	
Location	Age	Prevalence rate per 100,000 people (95% UI)	Incidence rate per 100,000 people (95% UI)	Prevalence rate per 100,000 people (95% UI)	Incidence rate per 100,000 people (95% UI)
Global	Age-standardized	68.94 (54.69,88.15)	503.25 (399.26,643.51)	40.24 (31.75,51.52)	293.74 (231.80,376.09)
	1-5 months	1586.55 (1258.91,2030.63)	11581.82 (9190.07,14823.59)	946.02 (747.47,1211.10)	6905.96 (5456.55,8841.03)
	6-11 months	1180.44 (936.78,1510.45)	8617.23 (6838.51,11026.3)	703.1 (555.41,900.12)	5132.6 (4054.48,6570.88)
	12-23 months	846 (671.29,1082.11)	6175.81 (4900.43,7899.43)	501.82 (396.27,642.46)	3663.3 (2892.79,4689.99)
	2-4 years	249.47 (197.89,318.93)	1821.16 (1444.57,2328.15)	144.15 (113.7,184.57)	1052.28 (830.04,1347.34)
	5-9 years	82.19 (65.18,105.04)	600.01 (475.83,766.81)	45.1 (35.5,57.75)	329.19 (259.17,421.58)
	10-14 years	18.1 (14.34,23.13)	132.13 (104.69,168.86)	10.1 (7.94,12.93)	73.71 (57.98,94.4)
	15-19 years	3.64 (2.89,4.67)	26.59 (21.11,34.08)	2.13 (1.67,2.72)	15.53 (12.21,19.89)
High-middle SDI	Age-standardized	54.47 (43.35,69.65)	397.62 (316.45,508.42)	31.03 (24.27,39.7)	226.55 (177.19,289.79)
Middle SDI	Age-standardized	66.98 (53.31,85.92)	488.96 (389.19,627.25)	35.97 (28.21,46.04)	262.56 (205.96,336.07)
Low-middle SDI	Age-standardized	90.69 (71.09,116.45)	662.01 (518.98,850.11)	40.91 (32.08,52.31)	298.61 (234.17,381.83)
Low SDI	Age-standardized	106.16 (82.12,136.79)	774.96 (599.51,998.54)	56.54 (45.1,72.47)	412.75 (329.22,529.05)
China	Age-standardized	36.75 (28.76,47.09)	268.25 (209.98,343.73)	13.92 (10.76,17.74)	101.59 (78.57,129.5)
	1-5 months	830.72 (650.09,1064.38)	6064.26 (4745.65,7769.94)	315.47 (244.01,402.15)	2302.93 (1781.3,2935.67)
	6-11 months	620.93 (485.9,795.57)	4532.77 (3547.1,5807.67)	235.92 (182.47,300.74)	1722.24 (1332.07,2195.41)
	12-23 months	447.87 (350.56,573.88)	3269.43 (2559.1,4189.32)	169.81 (131.34,216.46)	1239.62 (958.78,1580.18)
	2-4 years	133.51 (104.55,171.1)	974.63 (763.2,1249.03)	50.4 (38.98,64.24)	367.9 (284.55,468.97)
	5-9 years	44.44 (34.81,56.97)	324.44 (254.14,415.86)	16.82 (13.01,21.45)	122.81 (95,156.55)
	10-14 years	10.11 (7.92,12.96)	73.84 (57.84,94.64)	3.81 (2.94,4.85)	27.79 (21.49,35.42)
	15-19 years	2.14 (1.67,2.74)	15.62 (12.22,20.01)	0.8 (0.62,1.02)	5.83 (4.5,7.43)
India	Age-standardized	97.68 (76.08,125.82)	713.06 (555.41,918.52)	34.64 (27,44.18)	252.85 (197.09,322.53)
Japan	Age-standardized	37.18 (29.11,47.4)	271.42 (212.51,346.03)	6.42 (4.8,8.35)	46.86 (35.01,60.98)

For prevalence, the number of cases was highest in children aged 12–23 months, whereas the prevalence rate was again highest in infants aged 1–5 months (Table 2). Incidence followed a pattern similar to prevalence. These consistent rate-age disparities across three distinct metrics (mortality, prevalence, incidence) underscore that young infants, despite being a smaller population segment, bear a disproportionately high per-capita burden of pertussis globally.

Globally, age-standardized metrics (ASMR, ASDR, ASPR, and ASIR) all declined markedly from 1990 to 2023. In 1990, low-SDI regions already carried a substantially higher burden than high-middle-SDI regions (e.g., ASMR: 10.42 vs. 2.45 per 100,000). By 2023, although both improved, the low-SDI disadvantage persisted across all four metrics (Tables 1 and 2), reinforcing the strong association between socioeconomic development and pertussis burden.

### Global and SDI-level trends in childhood pertussis burden

**Fig 1:**
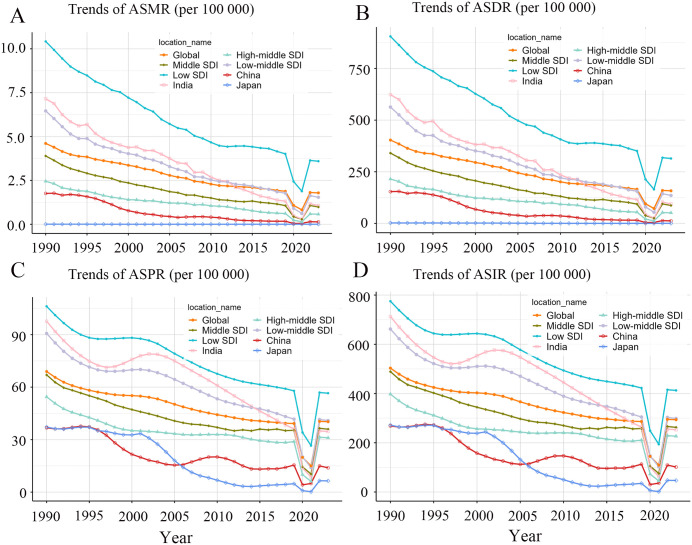
Global, SDI-regional, and national trends in ASMR, ASDR, ASPR, and ASIR of pertussis, with focus on China, India and Japan. (A) ASMR: age-standardized mortality rate; (B) ASDR: age-standardized DALYs rate; (C) ASPR: age-standardized prevalence rate; (D) ASIR: age-standardized incidence rate.

From 1990 to 2023, global ASMR, ASDR, ASPR, and ASIR for both genders followed steady downward trends (Fig 1). Across all SDI regions, declines were universal, but the steepest reductions occurred in low-SDI regions, an initially counterintuitive finding that likely reflects greater potential for improvement from a higher baseline (Fig 1). Notably, all four metrics showed a sharp, transient drop in 2020−2021, coinciding with COVID-19 pandemic disruption to health-care delivery and population mobility, a pattern evident worldwide and across all SDI regions. The AAPCs were significantly negative globally, with the most pronounced declines in high-middle-SDI regions (Table 3), indicating that while low-SDI regions caught up in speed of decline, high-middle-SDI regions achieved the largest annual reductions in absolute rate terms.

**Table 3:** The AAPC of mortality, DALYs, prevalence, and incidence rates in pertussis burden from 1990 to 2023.

		1990-2023 AAPC			
Location	Age	Deaths rate (95% UI)	DALYs rate (95% UI)	Prevalence rate (95% UI)	Incidence rate (95% UI)
Global	Age-standardized	−0.61 (−0.76,-0.3)	−0.61 (−0.76,-0.3)	−0.42 (−0.44,-0.39)	−0.42 (−0.44,-0.39)
	1-5months	−0.58 (−0.74,-0.23)	−0.58 (−0.74,-0.23)	−0.4 (−0.42,-0.38)	−0.4 (−0.42,-0.38)
	6-11months	−0.56 (−0.74,-0.21)	−0.55 (−0.74,-0.21)	−0.4 (−0.43,-0.38)	−0.4 (−0.43,-0.38)
	12-23months	−0.61 (−0.77,-0.28)	−0.61 (−0.77,-0.28)	−0.41 (−0.43,-0.38)	−0.41 (−0.43,-0.38)
	2-4 years	−0.63 (−0.79,-0.27)	−0.63 (−0.79,-0.27)	−0.42 (−0.44,-0.4)	−0.42 (−0.44,-0.4)
	5-9 years	−0.69 (−0.81,-0.48)	−0.69 (−0.81,-0.48)	−0.45 (−0.47,-0.42)	−0.45 (−0.47,-0.42)
	10-14 years	−0.61 (−0.77,-0.34)	−0.61 (−0.77,-0.34)	−0.44 (−0.46,-0.42)	−0.44 (−0.46,-0.42)
	15-19 years	−0.61 (−0.78,-0.25)	−0.61 (−0.78,-0.26)	−0.42 (−0.44,-0.39)	−0.42 (−0.44,-0.39)
China	Age-standardized	−0.92 (−0.99,-0.31)	−0.92 (−0.99,-0.34)	−0.62 (−0.65,-0.59)	−0.62 (−0.65,-0.59)
	1-5months	−0.93 (−0.99,-0.32)	−0.93 (−0.99,-0.35)	−0.62 (−0.65,-0.59)	−0.62 (−0.65,-0.59)
	6-11months	−0.95 (−0.99,-0.5)	−0.94 (−0.99,-0.51)	−0.62 (−0.65,-0.59)	−0.62 (−0.65,-0.59)
	12-23months	−0.91 (−0.99,-0.16)	−0.9 (−0.98,-0.2)	−0.62 (−0.65,-0.59)	−0.62 (−0.65,-0.59)
	2-4 years	−0.92 (−0.99,-0.27)	−0.91 (−0.99,-0.3)	−0.62 (−0.65,-0.59)	−0.62 (−0.65,-0.59)
	5-9 years	−0.92 (−0.99,-0.24)	−0.92 (−0.99,-0.26)	−0.62 (−0.65,-0.59)	−0.62 (−0.65,-0.59)
	10-14 years	−0.91 (−0.99,-0.05)	−0.91 (−0.99,-0.09)	−0.62 (−0.65,-0.59)	−0.62 (−0.65,-0.59)
	15-19 years	−0.91 (−0.99,0.03)	−0.9 (−0.99,-0.01)	−0.63 (−0.65,-0.59)	−0.63 (−0.65,-0.59)
High-middle SDI	Age-standardized	−0.77 (−0.91,-0.37)	−0.76 (−0.91,-0.37)	−0.43 (−0.45,-0.41)	−0.43 (−0.45,-0.41)
Middle SDI	Age-standardized	−0.75 (−0.89,-0.44)	−0.74 (−0.89,-0.44)	−0.46 (−0.48,-0.44)	−0.46 (−0.48,-0.44)
Low-middle SDI	Age-standardized	−0.76 (−0.91,-0.29)	−0.76 (−0.91,-0.29)	−0.55 (−0.57,-0.52)	−0.55 (−0.57,-0.52)
Low SDI	Age-standardized	−0.66 (−0.79,-0.41)	−0.65 (−0.79,-0.41)	−0.47 (−0.49,-0.44)	−0.47 (−0.49,-0.44)
India	Age-standardized	−0.85 (−0.98,0.33)	−0.85 (−0.98,0.3)	−0.65 (−0.67,-0.61)	−0.65 (−0.67,-0.61)
Japan	Age-standardized	−0.68 (−0.86,-0.28)	−0.8 (−0.84,-0.73)	−0.83 (−0.85,-0.8)	−0.83 (−0.85,-0.8)

### Children pertussis burden and gender disparities in China

In China, the absolute number of pertussis deaths and DALYs in 1990 and 2023 was highest among children aged 2–4 years. However, as observed globally, the rates of mortality, DALYs, prevalence, and incidence were consistently highest in infants aged 1–5 months in both years (Tables 1 and 2). This dissociation between absolute and rate-based burden where older children contribute more total cases but infants face highest individual risk, reappears at the national level. All four age-standardized metrics in China showed significant downward trends from 1990 to 2023 (Table 3).

**Fig 2:**
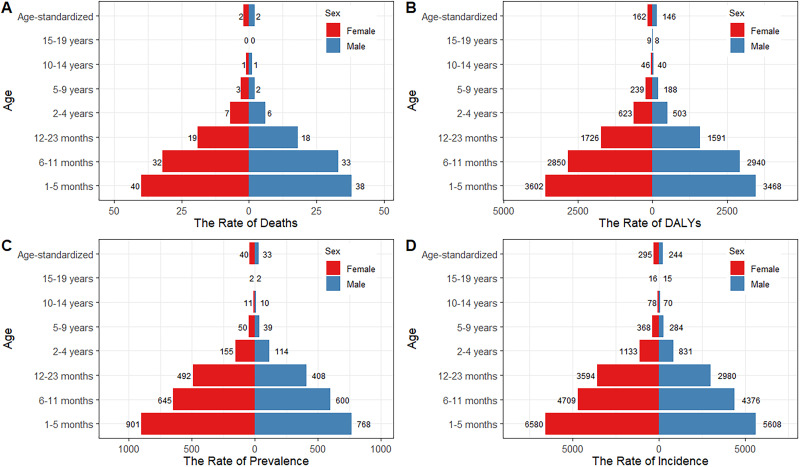
Comparison of the mortality, DALYs, prevalence, and incidence rates of pertussis between males and females in different age groups of Chinese children in 1990. Note that the values for females are slightly higher than those for males. (A) mortality; (B) DALYs; (C) prevalence; (D) incidence.

**Fig 3:**
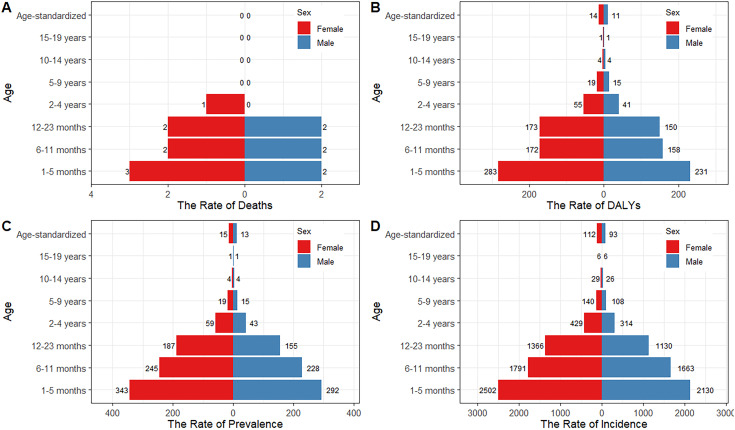
Comparison of the mortality, DALYs, prevalence, and incidence rates of pertussis between males and females in different age groups of Chinese children in 2023. Note that the values for females are slightly higher than those for males. (A) mortality; (B) DALYs; (C) prevalence; (D) incidence.

Figs 2 and 3 show that for both genders and across both time points, rates of all four metrics peaked in the 1–5-month age group and declined steadily with age. Female and male trajectories were largely paralleled, with females showing slightly higher rates in most age strata.

To interpret China’s performance, we benchmarked against global averages, India (similar population size but lower SDI), and Japan (higher SDI, regional neighbor). Chinese children had lower mortality, DALY, prevalence, and incidence rates than global and Indian counterparts, but consistently higher rates than Japanese children (S1–S6 Fig). This hierarchical gradient ranging from India, the global average, China to Japan aligns with corresponding SDI rankings, supporting the interpretation that socioeconomic development, healthcare access, and vaccination coverage likely drive between-country differences rather than unique biological or genetic factors.

### Joinpoint regression analysis of pertussis burden in China

**Fig 4:**
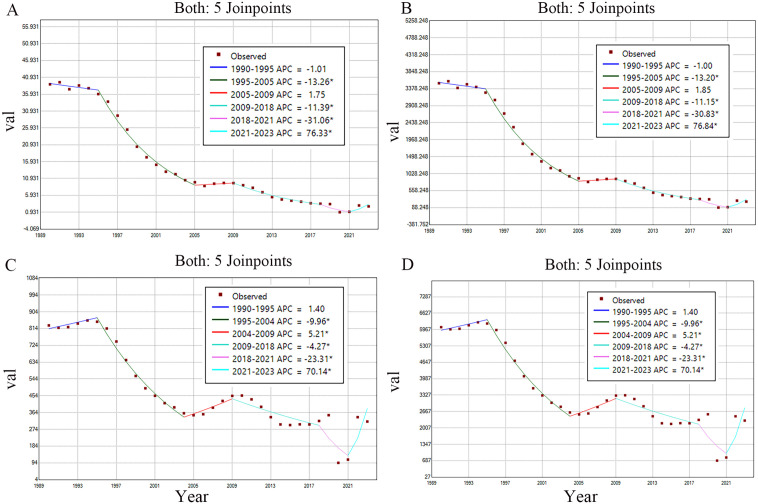
The APC of mortality, DALYs, prevalence, and incidence rates of pertussis in Chinese infants aged 1-5 months from 1990 to 2023. (A) mortality; (B) DALYs; (C) prevalence; (D) incidence.

Fig 4 displays Joinpoint regression results for both genders in China (1990–2023). The APC for mortality rate in infants aged 1–5 months declined significantly during three periods (1995–2005, 2009–2018, and 2018–2021), but then increased sharply from 2021 to 2023 (APC = 76.33, p < 0.05). DALY rates followed the same pattern. Prevalence and incidence rates also declined significantly in three periods, increased during 2004–2009 and again from 2021 to 2023 (APC = 70.14, p < 0.05). ASMR, ASDR, ASPR, and ASIR showed qualitatively similar up-and-down patterns (S7 Fig).

### Future burden of children pertussis in China

**Fig 5:**
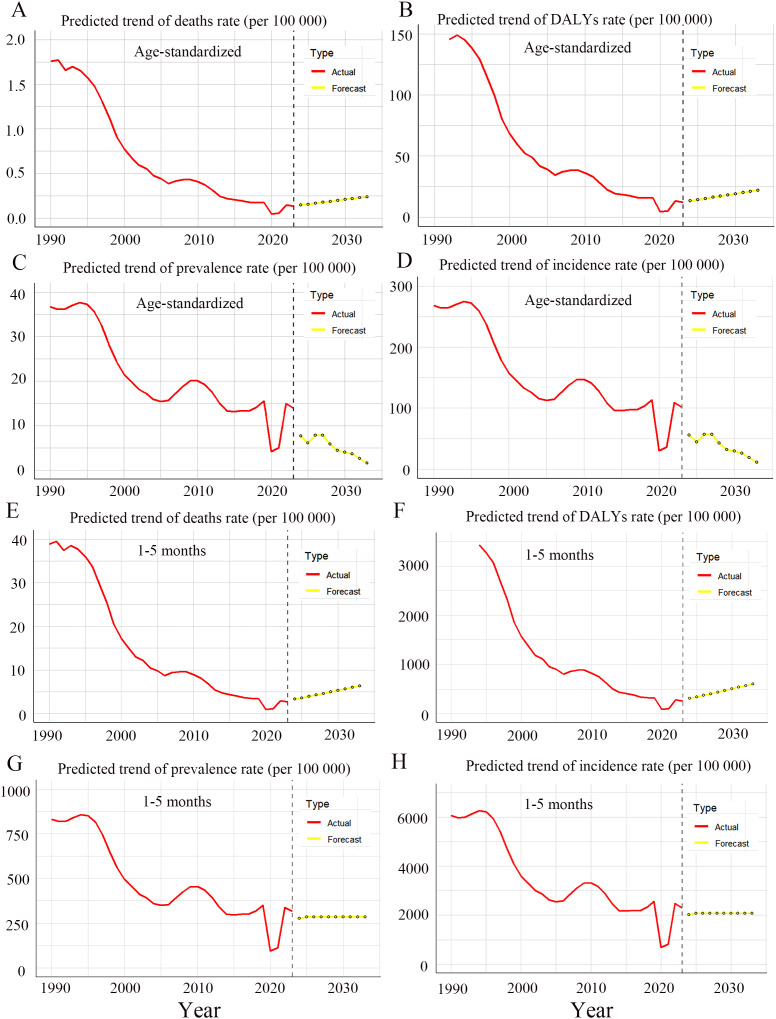
Forecast trajectories of pertussis mortality, DALYs, prevalence and incidence in China from 2024 to 2033. Red lines represent the actual trend of pertussis burden from 1990 to 2023; yellow lines represent the predicted trend for the next decade. (A) ASMR: age-standardized mortality rate; (B) ASDR: age-standardized DALYs rate; (C) ASPR: age-standardized prevalence rate; (D) ASIR: age-standardized incidence rate; (E) mortality rate in infants aged 1-5 months; (F) DALYs rate in infants aged 1-5 months; (G) prevalence rate in infants aged 1-5 months; (H) incidence rate in infants aged 1-5 months.

An ARIMA model was used to project pertussis burden trends over the next decade in China (Fig 5). In contrast to the historical declines, ASMR is projected to rise from 0.14 per 100,000 in 2024 to 0.24 per 100,000 population in 2033, and ASDR from 13.23 in 2024 to 21.98 per 100,000 population in 2033 (Fig 5). Conversely, ASPR and ASIR are predicted to decline markedly. Among infants aged 1–5 months, mortality and DALY rates are projected to follow the same upward trajectory as the age-standardized metrics, while prevalence and incidence in this same age group will edge slightly upward (from 277.59 to 284.04 per 100,000 for prevalence). Together, these projections indicate that although overall prevalence may fall, young infants will face a persistent or even growing relative risk. Globally, by contrast, mortality and DALY rates are expected to continue declining, while prevalence and incidence increase slightly over the next decade, whether measured as age-standardized estimates or among infants aged 1–5 months (S8 Fig). These findings further demonstrate that infants aged 1–5 months bear a disproportionately high burden of pertussis worldwide.

## Discussion

Despite high global vaccine coverage, pertussis remains the leading vaccine-preventable cause of childhood morbidity worldwide [1,2]. Infants under 1 year of age bear a grossly disproportionate share of this burden, with pertussis incidence, mortality, and DALYs surpassing those of all other pediatric cohorts [3]. Our finding identifies divergent age-based distributions, total mortality peaks among children aged 2–4 years, while mortality rates remain consistently highest in infants aged 1–5 months, highlighting a marked mismatch between aggregate disease burden and individual-level disease risk. Such epidemiological divergence implies that older children drive overall case volumes, largely stemming from waning vaccine-induced immunity and intensified interpersonal contact at school; in contrast, young infants suffer elevated individual risk as they are ineligible to finish the primary immunization schedule due to young age. From the public health perspective, these findings underscore the necessity of dual-targeted interventions: optimizing booster immunization for school-age children and advancing early protective strategies for vulnerable young infants.

The situation is deteriorating rapidly in Europe, where pertussis cases, especially severe illness and deaths among young infants, continue to climb [14]. French nationwide data also confirm that pertussis remains a persistent public health challenge, especially in the first year of life [2]. In Central Europe, pertussis resurged dramatically in 2024 [15]. Slovakia reported 7,043 cases compared with only 216 in 2023. Neighboring countries also experienced sharp increases, including the Czech Republic, Poland, Austria, and Ukraine. Notably, over half of the cases occurred in appropriately vaccinated individuals, highlighting waning vaccine-induced immunity [15]. A similar trend has emerged in Latin America. The incidence per million in Argentina, Chile, Peru and Uruguay rose sharply between 2021 and 2023, with the majority of cases occurring in infants [16]. In Brazil, pertussis cases surged 34.4-fold in 2024–7,438 cases, accompanied by 30 deaths and a 3.1-fold rise in hospitalizations, with infections increasingly affecting older age groups [17]. In East Asia, Japan experienced a delayed but massive outbreak in 2025, recording 60,826 cases by July, a sharp rise from 4,054 cases in 2024. Notably, 56.6% of cases occurred in adolescents aged 5–19 years, suggesting that the lack of routine booster vaccination may be a key driver [18]. By contrast, in low-SDI regions such as Somalia, pertussis still imposes a heavy burden, underscoring the imperative for sustained financial and medical support [3]. These regional signals are almost certainly underestimates: sub-optimal PCR capacity, inconsistent case definitions, and pandemic-disrupted surveillance networks have created detection gaps that mask the true reservoir of transmission. Therefore, strengthening surveillance systems encompassing molecular diagnostics, standardized case reporting, and sentinel surveillance networks is urgently needed to accurately capture the true burden and guide timely public health responses. With the incidence peaking in infants under 1 year, higher vaccination coverage, rapid diagnostics, and aggressive complication-prevention are now imperative.

Notably, China is mirroring the global epidemiologic shift. After a steady decline from 1990 through 2023, the national pertussis incidence has risen sharply in the last few years. In southwest Sichuan, pertussis has overtaken measles, rubella, and mumps to become the top notifiable respiratory infection. Its incidence soared to 67.12 per 100,000 people in 2024 from just 0.53 per 100,000 people in 2005 [5]. Zhejiang province reported that the incidence of pediatric pertussis among 0–18-year-olds increased from 14.22 per 100,000 people in 2023 to 512.56 per 100,000 people in 2024, mainly driven by 3–9-year-olds [19]. In 2021–2022, Yiwu, Zhejiang Province, had a pertussis-hospitalization rate of 2.50 per 100,000 people, while Yongcheng, Henan Province, reached a rate of 7.90 per 100,000 people. In both cities, infants aged < 1 year had the highest hospitalization rate, followed by the 4–5-year-old group [20]. In Hubei, children aged 5–9 years were the most affected, whereas infants aged 0–2 months were hospitalized most severely [7].

The pertussis resurgence observed globally and in China can be attributed to multiple interacting factors, including enhanced case ascertainment and reporting, the emergence of mutant B. pertussis strains capable of immune escape from acellular and whole-cell vaccines, reduced susceptibility to macrolides, and waning natural or vaccine-induced immunity [21,22]. In the present study, we observed a sharp decline in pertussis incidence globally and in China from 2020 to 2021, followed by a steady recovery from 2022 to 2023. This epidemiological shift is likely driven by COVID-19-associated non-pharmaceutical interventions (NPIs), including mask-wearing, regular hand hygiene and social distancing, alongside subsequent accumulation of immunity debt [23]. Despite good consistency between our temporal trends and the above hypothesis, dedicated external analyses are needed to confirm causal links between regional NPI stringency and post-pandemic pertussis resurgence.

Our ARIMA predictive model indicated that both ASMR and ASDR for pertussis in China will edge upward over the next decade, whereas the age-standardized prevalence and incidence rates are expected to fall steeply. Notably, infants aged 1–5 months exhibit a significantly higher prevalence and incidence compared to the national age-standardized figures, and this gap is predicted to widen. These projections inform vaccination policies by endorsing maternal immunization for newborn protection, advocating earlier initial DTaP dosing to shorten infant vulnerability periods, recommending routine preschool and school-age booster shots to curb community spread, and mandating enhanced surveillance for real-time vaccine efficacy and variant monitoring.

In contrast, a comprehensive modeling by Xu *et al.* predicts that the global adolescent pertussis burden will reach its peak in 2022, followed by a gradual decline until 2046 [24]. This trajectory diverges sharply from our findings for Chinese infants under 6 months. Instead of reflecting conflicting results, this discrepancy underscores a core epidemiological discrepancy: pertussis among adolescents and young infants arises from distinct immunologic and behavioral drivers, namely waning vaccine-induced immunity in teenagers and incomplete primary vaccination schedules in infants, mandating differentiated prevention strategies.

Vaccination remains the most effective preventive measure for pertussis. Before 2025, China’s immunization strategy included four doses of diphtheria, tetanus, and acellular pertussis (DTaP) vaccine at 3, 4, 5, and 18 months [19]. In children under 2 years of age, the overall vaccine effectiveness against pertussis disease or hospitalization demonstrated an increase with a higher number of administered co-purified DTaP vaccine doses. Directly derived from our finding that infants aged 1–5 months face the highest per-capita risk and that this age group will continue to bear a disproportionate burden over the next decade, it is important to reinforce timely and complete DTaP vaccination for age-appropriate children and to investigate the possibility of administering the first dose at 2 months of age to minimize the risk of pertussis disease and hospitalization in infants [25]. Additionally, expediting rollout of maternal immunization programs may substantially reduce pertussis-associated neonatal morbidity and mortality.

Global diphtheria-tetanus-pertussis vaccination coverage nearly doubled between 1980 and 2023. However, in many countries and territories, coverage gains slowed between 2010 and 2019. Furthermore, the COVID-19 pandemic exacerbated these challenges. Since 2020, the global rates for these vaccines have declined sharply, and as of 2023, they still have not returned to pre-COVID-19 pandemic levels [26]. Given our projection of rising mortality and DALY rates in Chinese infants, a booster dose for older children and adults is urgently needed. Moreover, the durability, safety, and cost-effectiveness of currently available vaccines should be re-evaluated, particularly in light of waning immunity patterns documented in other high-burden settings.

Despite the considerable strengths of our analysis, there were several limitations. First, as with all GBD studies, our estimates rely on modeled reconstructions rather than direct measurements. Any biases in the underlying assumptions (e.g., sparse surveillance data, cause-specific mortality attribution) will propagate into our findings. Second, our ARIMA-based forecasts are inherently uncertain, particularly over longer horizons and given unstable post-pandemic trends. The projected increases should be interpreted as conditional scenarios, not definitive predictions. Third, the pertussis case definition remains contingent upon laboratory confirmation. Yet individuals with non-specific or only moderately severe cough frequently evade clinician sampling, especially where health-care access is uneven. This diagnostic gap almost certainly creates a hidden reservoir of infections that are invisible to routine surveillance systems, leading to a non-trivial downward bias in incidence and mortality estimates. Fourth, the GBD 2023 release is not stratified by housing density, household crowding index, or sub-national administrative units. These social determinants demonstrably modulate transmission networks through differential contact rates and vaccination coverage.

In summary, childhood pertussis deaths, DALYs, prevalence and incidence have declined steeply worldwide and in China over the past three decades, with consistent downward trends in ASMR, ASDR, ASPR and ASIR across all SDI regions. In China, each indicator reaches its peak at 1–5 months of age and decreases steadily as age increases. Forecasts suggest that this same age group will experience a modest yet continuous rise in mortality, DALYs, prevalence and incidence over the next decade, highlighting the disproportionate and persistent burden on the youngest infants. It further emphasizes the urgent necessity for targeted prevention and early intervention strategies to protect this particularly vulnerable population.

## Supporting information

S1 Table The number of mortality and DALYs in pertussis burden for both genders between 1990 and 2023. (DOCX)

S2 Table The number of prevalence and incidence in pertussis burden for both genders between 1990 and 2023. (DOCX)

S1 Fig Comparison of the mortality, DALYs, prevalence, and incidence rates of global pertussis in males and females of different age groups in 1990. (A) mortality; (B) DALYs; (C) prevalence; (D) incidence.
(TIF)

S2 Fig Comparison of the mortality, DALYs, prevalence, and incidence rates of global pertussis in males and females of different age groups in 2023. (A) mortality; (B) DALYs; (C) prevalence; (D) incidence.
(TIF)

S3 Fig Comparison of the mortality, DALYs, prevalence, and incidence rates of pertussis in males and females of different age groups in India in 1990. (A) mortality; (B) DALYs; (C) prevalence; (D) incidence.
(TIF)

S4 Fig Comparison of the mortality, DALYs, prevalence, and incidence rates of pertussis in males and females of different age groups in India in 2023. (A) mortality; (B) DALYs; (C) prevalence; (D) incidence.
(TIF)

S5 Fig Comparison of the mortality, DALYs, prevalence, and incidence rates of pertussis in males and females of different age groups in Japan in 1990. (A) mortality; (B) DALYs; (C) prevalence; (D) incidence.
(TIF)

S6 Fig Comparison of the mortality, DALYs, prevalence, and incidence rates of pertussis in males and females of different age groups in Japan in 2023. (A) mortality; (B) DALYs; (C) prevalence; (D) incidence.
(TIF)

S7 Fig The APC of age-standardized mortality, DALYs, prevalence, and incidence rates of pertussis in China from 1990 to 2023. (A) mortality; (B) DALYs; (C) prevalence; (D) incidence.
(TIF)

S8 Fig Forecast trajectories of global pertussis mortality, DALYs, prevalence and incidence from 2024 to 2033. Red lines represent the true trend of pertussis burden during 1990–2023; yellow lines represent the predicted trend for the coming decade. (A) ASMR: age-standardized mortality rate; (B) ASDR: age-standardized DALYs rate; (C) ASPR: age-standardized prevalence rate; (D)ASIR: age-standardized incidence rate; (E) mortality rate in infants with 1–5 months; (F) DALYs rate in infants with 1–5 months; (G) prevalence rate in infants with 1–5 months; (H) incidence rate in infants with 1–5 months.
(TIF)

S1 File (ZIP)

S2 File (ZIP)

S3 File (ZIP)
