# Supplementary material for: Temporal trends in children pertussis burden in China and worldwide from 1990 to 2023: An analysis of the Global Burden of Disease Study 2023
Source: PLoS One. 2026 Jul 27;21(7):e0354164. doi: 10.1371/journal.pone.0354164 (PMC13405100; doi:10.1371/journal.pone.0354164)
Supplement: S1 Table — (DOCX) [file pone.0354164.s001.docx]

Table S1. The number of mortality and DALYs in pertussis burden for both genders between 1990 and 2023

|  |  | 1990 |  | 2023 |  |
| --- | --- | --- | --- | --- | --- |
| Location | Age | Deaths number (95% UI) | DALYs number (95% UI) | Deaths number (95% UI) | DALYs number (95% UI) |
| Global | 1-5 months | 58210.25 (31037.58,98847.23) | 5267332.23 (2815814.54,8923184.96) | 24206.76 (13771.29,41798.67) | 2197774.93 (1260167.3,3777422.55) |
|  | 6-11 months | 46936.73 (24286.1,80658.49) | 4228790.32 (2203143.25,7250078.36) | 20795.2 (12068.39,35601.97) | 1879180.53 (1096008.32,3199664.53) |
|  | 12-23 months | 62722.64 (31367.76,107932.19) | 5607709.89 (2820534.77,9621091.41) | 24660.31 (14317.98,40529.69) | 2215792.88 (1294348.19,3628472.62) |
|  | 2-4 years | 65820.86 (32444.29,110116.20) | 5758647.17 (2859172.12,9612397.27) | 25953.68 (14786.20,43587.41) | 2280260.59 (1308708.29,3810829.33) |
|  | 5-9 years | 36047.89 (18140.02,63640.12) | 3018430.65 (1522508.46,5312032.99) | 13445.15 (7753.48,21309.28) | 1131403.98 (659779.66,1783142.46) |
|  | 10-14 years | 6518.56 (3396.37,11484.97) | 511830.43 (268203.99,898994.14) | 3239.53 (1866.2,5356.14) | 254876.98 (147505.54,421195.29) |
|  | 15-19 years | 1341.02 (681.56,2414.18) | 98268.32 (50862.38,176294.09) | 651.89 (369.23,1091.16) | 47981.87 (27478.19,80187.80) |
| China | 1-5 months | 3662.28 (311.01,10536.19) | 332653.85 (31103.59,949635.83) | 105.67 (11.53,297.03) | 10115.04 (1670.68,27313.25) |
|  | 6-11 months | 3513.32 (323.59,9513.23) | 317214.00 (31742.09,853267.47) | 81.86 (10.79,198.68) | 7881.39 (1516.24,18240.27) |
|  | 12-23 months | 4062.59 (392.66,10518.61) | 364811.92 (38974.93,936562.58) | 179.10 (22479.37) | 16756.38 (2838.69,43217.53) |
|  | 2-4 years | 4251.44 (401.55,11151.00) | 373460.56 (38561.29,973271.39) | 221.16 (27.77,563.44) | 20241.28 (3505.90,50568.58) |
|  | 5-9 years | 2652.87 (248.34,7218.85) | 222237.73 (22526.97,600281.27) | 196.10 (22.13,500.89) | 17070.95 (2704.39,42521.69) |
|  | 10-14 years | 554.38 (47.34,1621.17) | 43544.53 (4206.65,126341.66) | 43.69 (5.1,117.3) | 3561.63 (549.12,9263.09) |
|  | 15-19 years | 146.18 (12.39,428.45) | 10729.36 (1019.64,31214.57) | 8.5 (0.84,23.85) | 650 (93.77,1765.43) |
