# Supplementary material for: Temporal trends in children pertussis burden in China and worldwide from 1990 to 2023: An analysis of the Global Burden of Disease Study 2023
Source: PLoS One. 2026 Jul 27;21(7):e0354164. doi: 10.1371/journal.pone.0354164 (PMC13405100; doi:10.1371/journal.pone.0354164)
Supplement: S2 Table — (DOCX) [file pone.0354164.s002.docx]

Table S2. The number of prevalence and incidence in pertussis burden for both sexes between 1990 and 2023

|  |  | 1990 |  | 2023 |  |
| --- | --- | --- | --- | --- | --- |
| Location | Age | Prevalence number  (95% UI) | Incidence number  (95% UI) | Prevalence number (95% UI) | Incidence number  (95% UI) |
| Global | 1-5 months | 854402.15 (677960.49,1093550.64) | 6237135.66 (4949111.54,7982919.65) | 500806.53 (395698.51,641134.33) | 3655887.68 (2888599.14,4680280.65) |
|  | 6-11 months | 734858.05 (583172.39,940297.97) | 5364463.79 (4257158.46,6864175.21) | 436620.47 (344907.04,558972.68) | 3187329.42 (2517821.39,4080500.56) |
|  | 12-23 months | 1044257.14 (828605.01,1335700.58) | 7623077.12 (6048816.54,9750614.2) | 630402.33 (497808.94,807081.49) | 4601937.03 (3634005.27,5891694.85) |
|  | 2-4 years | 910219.8 (722000.5,1163617.29) | 6644604.55 (5270603.67,8494406.22) | 566172.3 (446598.01,724926.38) | 4133057.75 (3260165.48,5291962.61) |
|  | 5-9 years | 479117.28 (379955.88,612302.37) | 3497556.16 (2773677.89,4469807.31) | 315910.34 (248708.49,404565.22) | 2306145.48 (1815571.99,2953326.13) |
|  | 10-14 years | 97163.88 (76990.62,124176.3) | 709296.33 (562031.55,906486.96) | 68908.08 (54205.21,88249) | 503028.99 (395698.06,644217.67) |
|  | 15-19 years | 18968.94 (15056.77,24310.66) | 138473.25 (109914.4,177467.79) | 13711.7 (10778.49,17560.46) | 100095.44 (78682.98,128191.34) |
| China | 1-5 months | 78260.61 (61243.7,100272.87) | 571302.44 (447078.98,731991.95) | 12507.67 (9674.63,15944.26) | 91305.99 (70624.82,116393.06) |
|  | 6-11 months | 67979.93 (53197.31,87100.16) | 496253.51 (388340.36,635831.16) | 11285.66 (8728.89,14386.3) | 82385.31 (63720.91,105019.97) |
|  | 12-23 months | 98731.99 (77280.91,126511.17) | 720743.52 (564150.63,923531.57) | 17715.31 (13701.97,22582.39) | 129321.78 (100024.41,164851.43) |
|  | 2-4 years | 89066.65 (69745.3,114142.84) | 650186.56 (509140.71,833242.75) | 21306.38 (16479.18,27159.85) | 155536.57 (120298.05,198266.88) |
|  | 5-9 years | 46469.09 (36400.63,59562.48) | 339224.35 (265724.59,434806.12) | 17019.42 (13165.2,21695.95) | 124241.75 (96105.99,158380.43) |
|  | 10-14 years | 10372.98 (8125.07,13295.09) | 75722.76 (59312.98,97054.13) | 3378.88 (2612.99,4307.26) | 24665.81 (19074.8,31443) |
|  | 15-19 years | 2717.44 (2126.13,3481.38) | 19837.33 (15520.75,25414.07) | 642.93  (496.88,819.85) | 4693.36 (3627.25,5984.93) |
