# Supplementary figures and images for: Temporal trends in children pertussis burden in China and worldwide from 1990 to 2023: An analysis of the Global Burden of Disease Study 2023
Source: PLoS One. 2026 Jul 27;21(7):e0354164. doi: 10.1371/journal.pone.0354164 (PMC13405100; doi:10.1371/journal.pone.0354164)

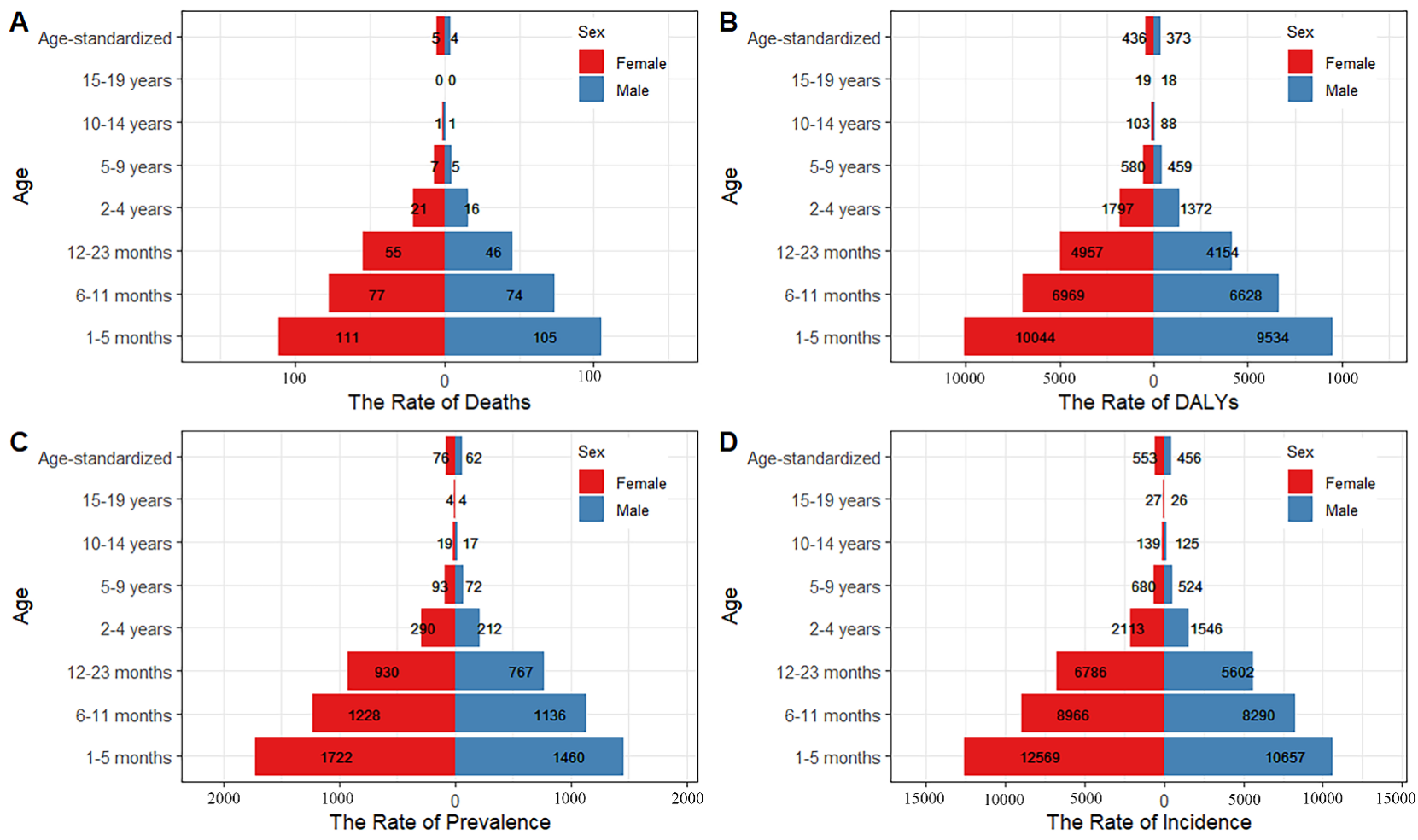

Supplement: S1 Fig — (A) mortality; (B) DALYs; (C) prevalence; (D) incidence. (TIF) [file pone.0354164.s003.tif]

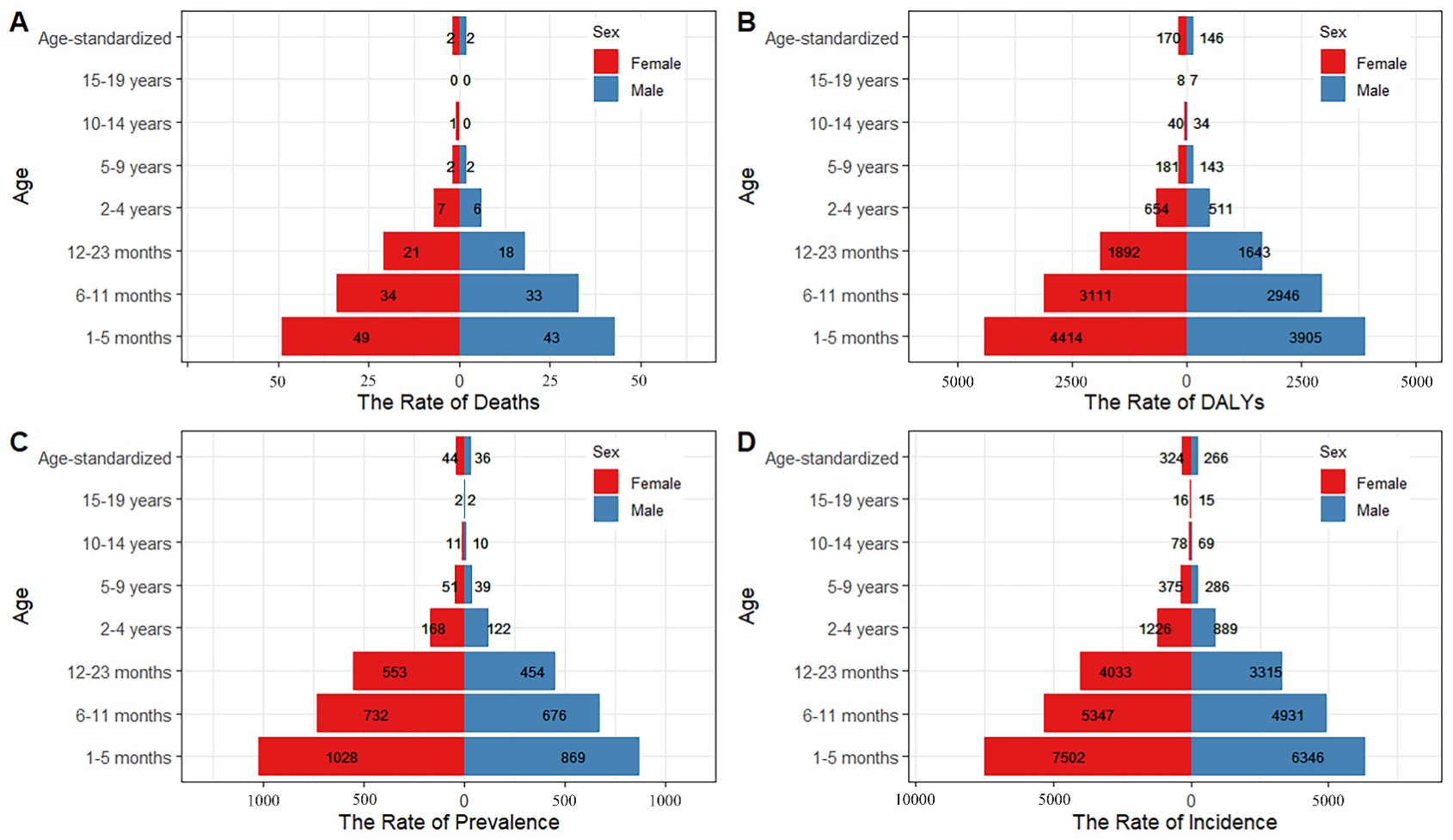

Supplement: S2 Fig — (A) mortality; (B) DALYs; (C) prevalence; (D) incidence. (TIF) [file pone.0354164.s004.tif]

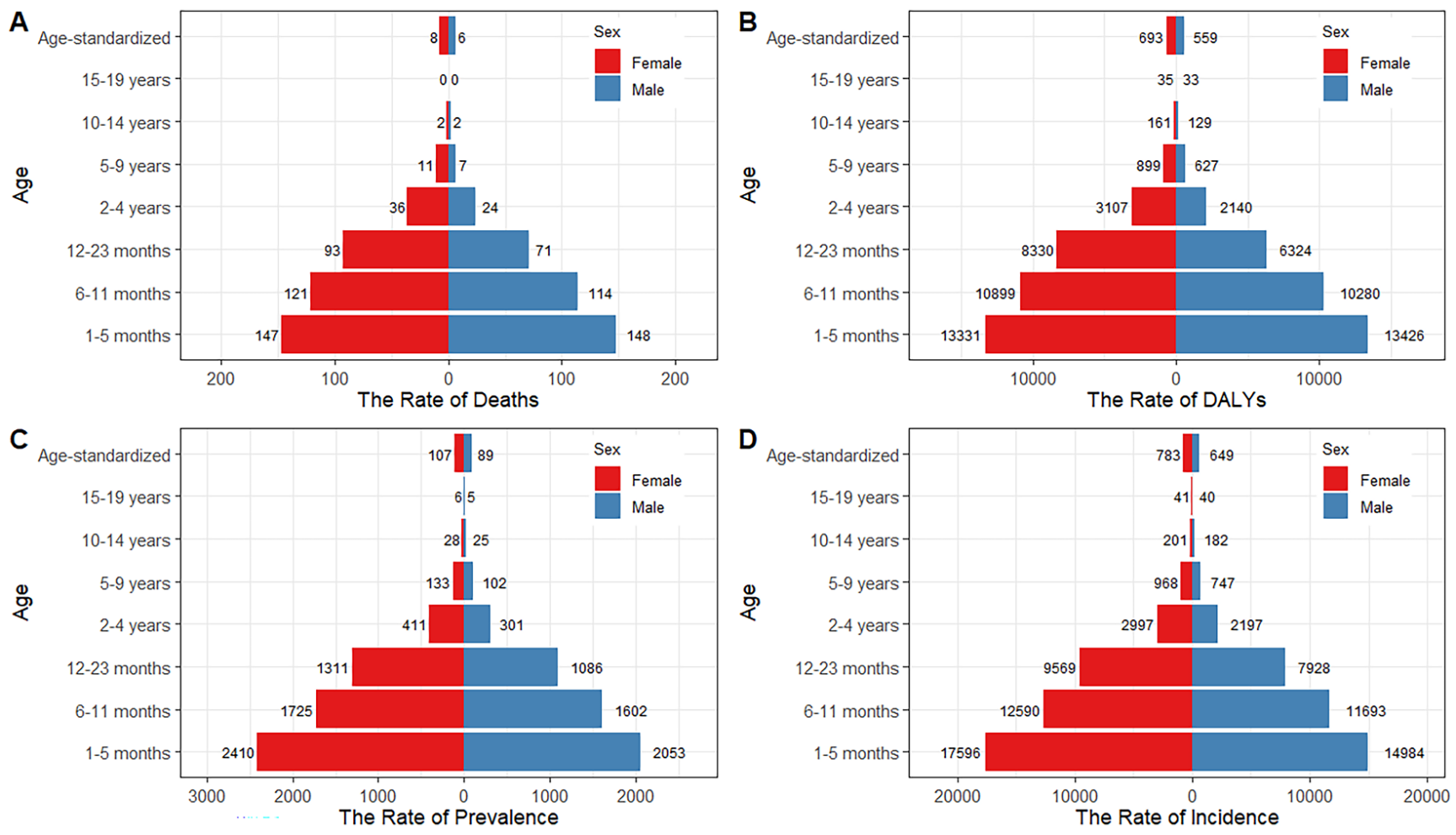

Supplement: S3 Fig — (A) mortality; (B) DALYs; (C) prevalence; (D) incidence. (TIF) [file pone.0354164.s005.tif]

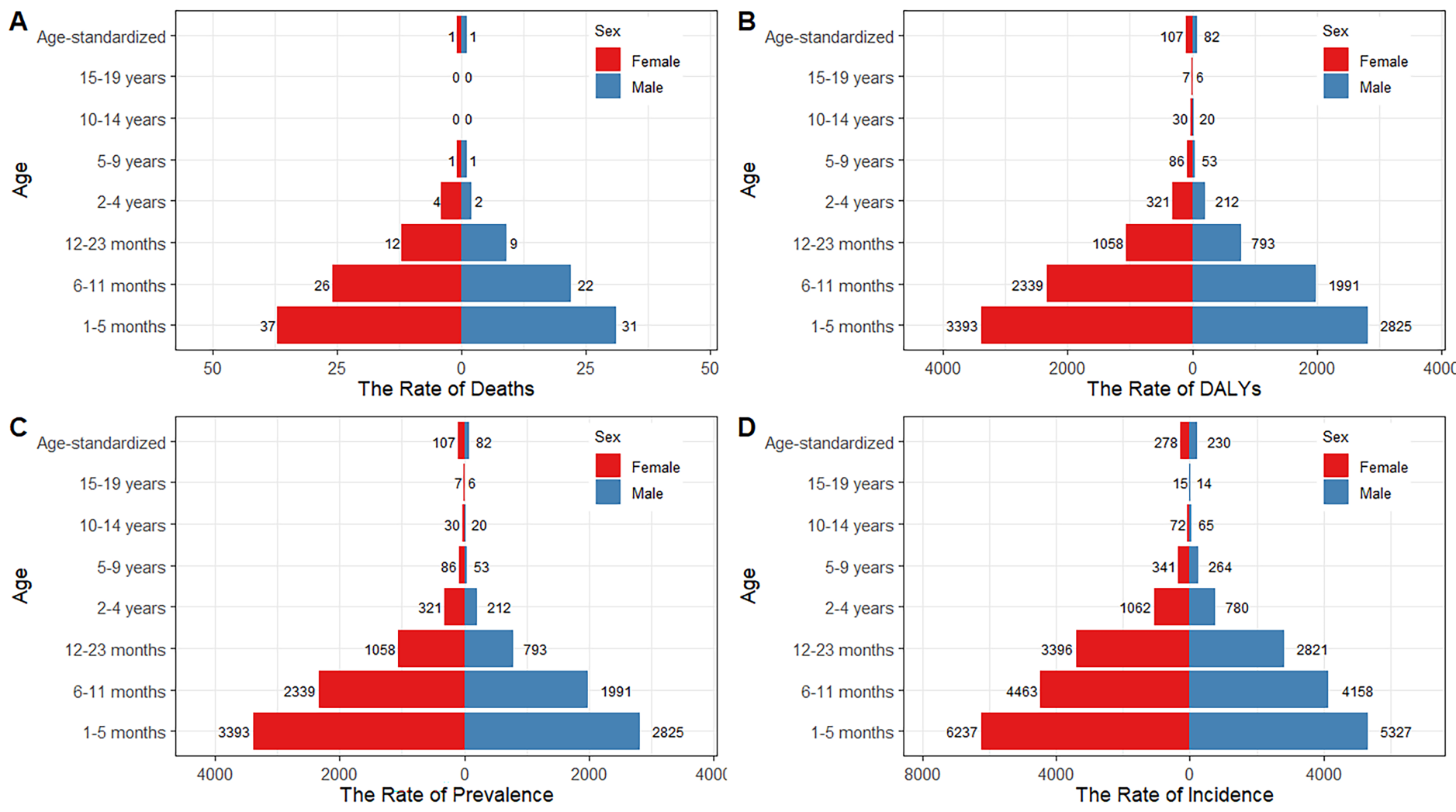

Supplement: S4 Fig — (A) mortality; (B) DALYs; (C) prevalence; (D) incidence. (TIF) [file pone.0354164.s006.tif]

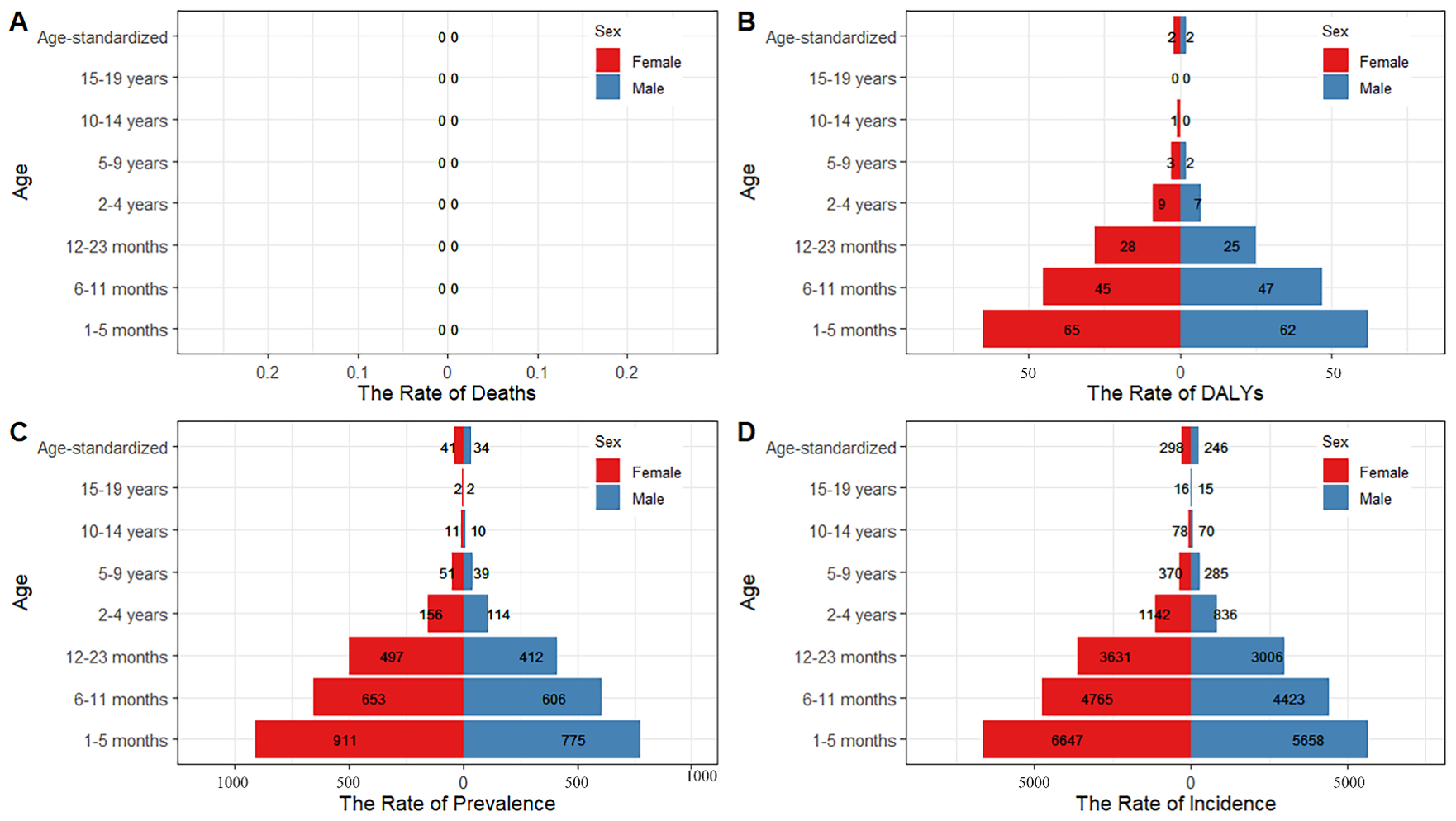

Supplement: S5 Fig — (A) mortality; (B) DALYs; (C) prevalence; (D) incidence. (TIF) [file pone.0354164.s007.tif]

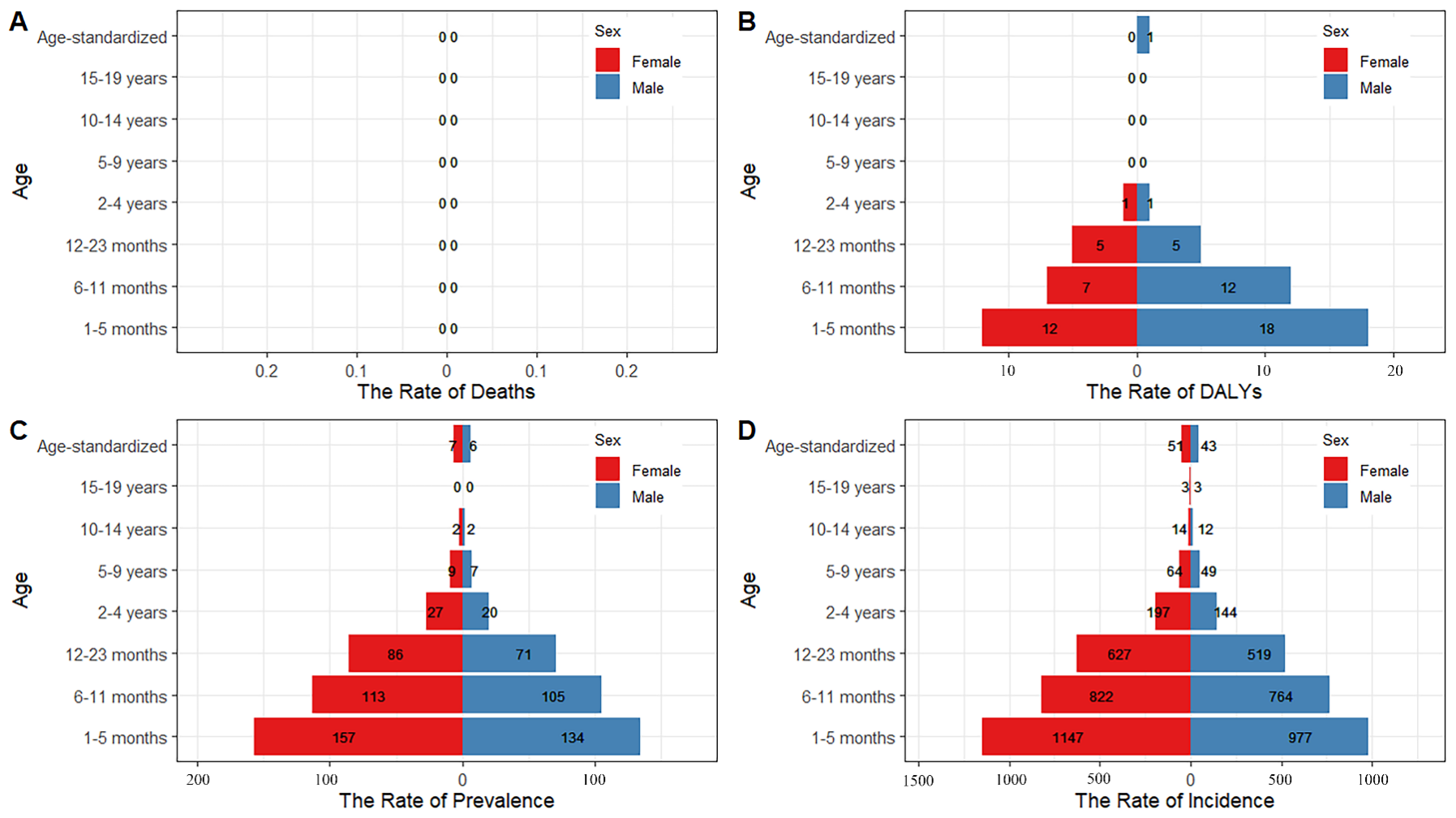

Supplement: S6 Fig — (A) mortality; (B) DALYs; (C) prevalence; (D) incidence. (TIF) [file pone.0354164.s008.tif]

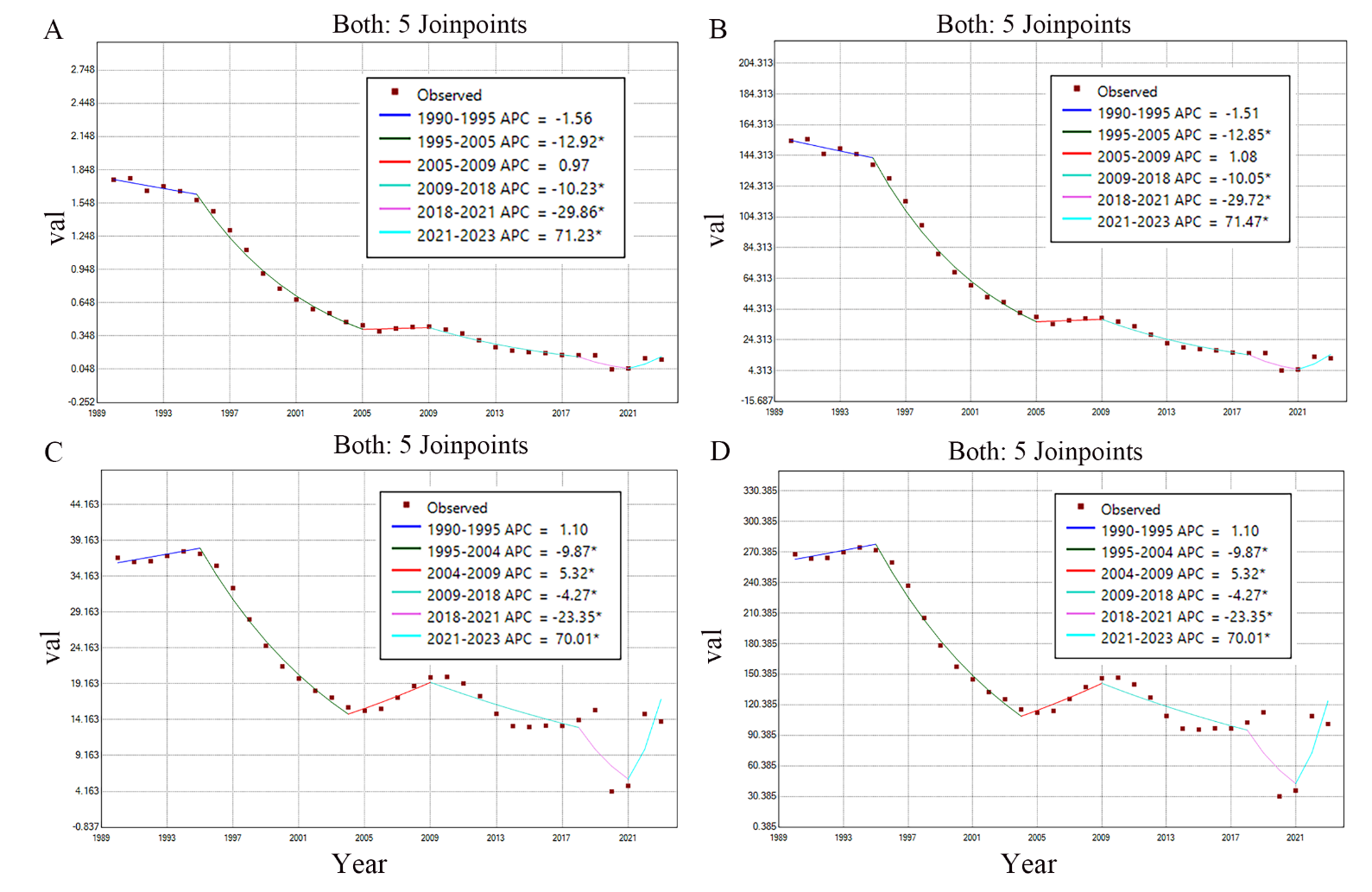

Supplement: S7 Fig — (A) mortality; (B) DALYs; (C) prevalence; (D) incidence. (TIF) [file pone.0354164.s009.tif]

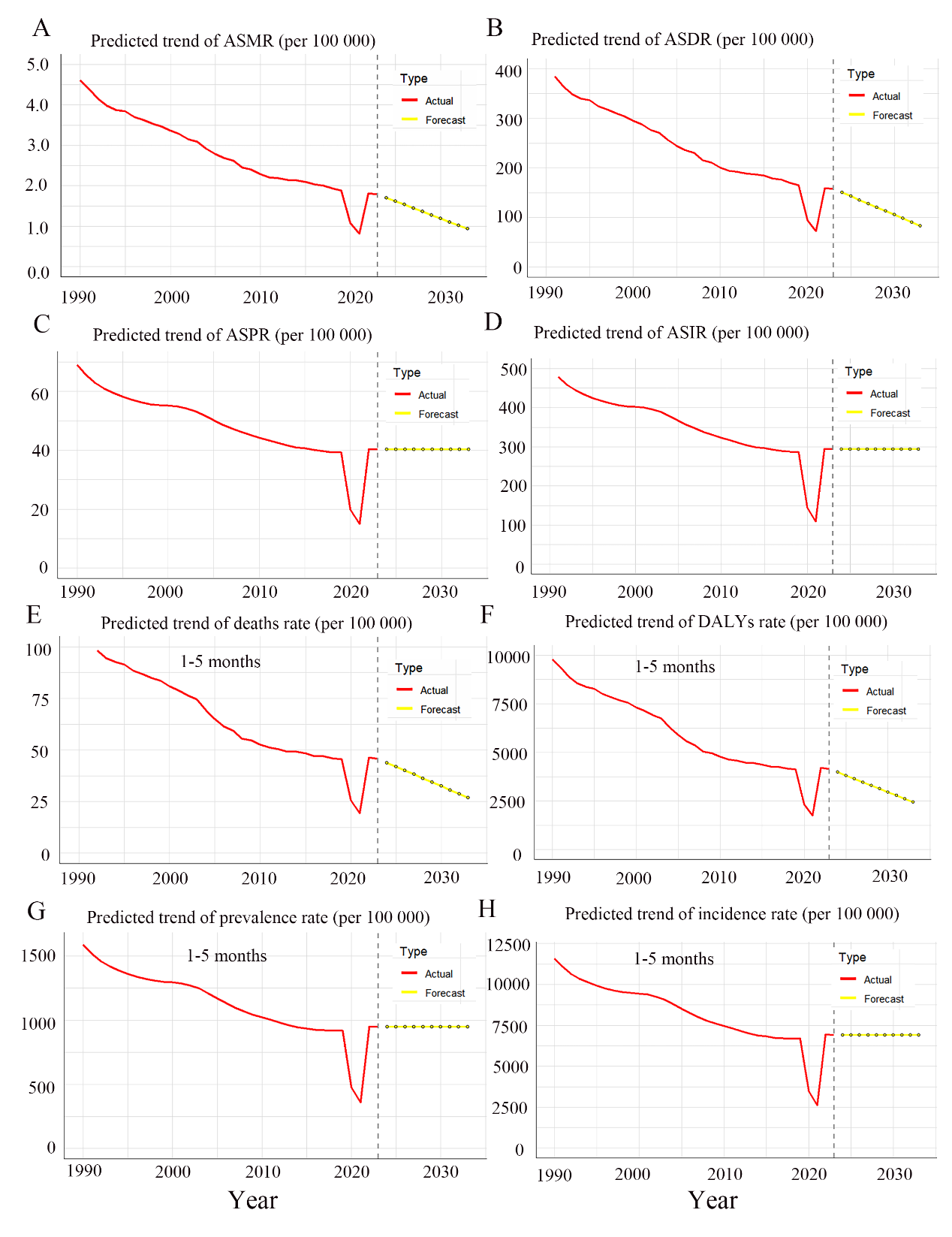

Supplement: S8 Fig — Red lines represent the true trend of pertussis burden during 1990–2023; yellow lines represent the predicted trend for the coming decade. (A) ASMR: age-standardized mortality rate; (B) ASDR: age-standardized DALYs rate; (C) ASPR: age-standardized prevalence rate; (D)ASIR: age-standardized incidence rate; (E) mortality rate in infants with 1–5 months; (F) DALYs rate in infants with 1–5 months; (G) prevalence rate in infants with 1–5 months; (H) incidence rate in infants with 1–5 months. (TIF) [file pone.0354164.s010.tif]
